# Renal Proximal Tubular Cells: A New Site for Targeted Delivery Therapy of Diabetic Kidney Disease

**DOI:** 10.3390/ph15121494

**Published:** 2022-11-30

**Authors:** Hao Li, Wenni Dai, Zhiwen Liu, Liyu He

**Affiliations:** Department of Nephrology, The Second Xiangya Hospital of Central South University, Hunan Key Laboratory of Kidney Disease and Blood Purification, Changsha 410011, Hunan, China

**Keywords:** diabetic nephropathies, drug delivery systems, kidney tubules, proximal, molecular targeted therapy, diabetes mellitus

## Abstract

Diabetic kidney disease (DKD) is a major complication of diabetes mellitus (DM) and the leading cause of end-stage kidney disease (ESKD) worldwide. A significant number of drugs have been clinically investigated for the treatment of DKD. However, a large proportion of patients still develop end-stage kidney disease unstoppably. As a result, new effective therapies are urgently needed to slow down the progression of DKD. Recently, there is increasing evidence that targeted drug delivery strategies such as large molecule carriers, small molecule prodrugs, and nanoparticles can improve drug efficacy and reduce adverse side effects. There is no doubt that targeted drug delivery strategies have epoch-making significance and great application prospects for the treatment of DKD. In addition, the proximal tubule plays a very critical role in the progression of DKD. Consequently, the purpose of this paper is to summarize the current understanding of proximal tubule cell-targeted therapy, screen for optimal targeting strategies, and find new therapeutic approaches for the treatment of DKD.

## 1. Introduction

Diabetic kidney disease (DKD) is one of the main complications of diabetes mellitus (DM) and is also the main cause of end-stage kidney disease (ESKD). The prevalence of ESKD in the general adult population in mainland China is 10.8%. With the rapid development of the world economy and the increasingly serious aging of the population, the incidence of ESKD is increasing year by year [1,2]. At present, there are approximately 100 million DKD patients in the world, resulting in huge medical costs and a huge economic burden [3]. The main treatment methods for DKD are blood pressure control and blood glucose control, and commonly used drugs include angiotensin-converting enzyme inhibitors (ACEI), angiotensin II receptor blockers (ARB), mineralocorticoid receptor antagonists (MRAs), dipeptidyl peptidase 4(DPP-4) inhibitor glucagon-like peptide-1 (GLP-1) receptor agonist, and sodium glucose cotransporter 2(SGLT2) inhibition [4,5,6,7,8,9]. In addition, in recent years, papers have reported new drugs for the treatment of DKD, such as: luteolin attenuates, protein arginine methyltranferase-1, stanniocalcin-1 (STC-1), adiponectin, and microRNA-122-5p [10,11,12,13,14]. However, these drugs make it difficult for them to control the progression of DKD due to their side effects or difficulties in achieving effective accumulation in the kidney. In such cases, many patients inevitably develop ESKD. Therefore, new therapeutic methods are urgently needed to be developed and applied to DKD.

Targeted drug delivery therapy refers to the use of the specific carrier to wrap active pharmaceutical ingredients [15] that can be transported to designated organs or cells via the carrier. In this way, its local drug concentration can be greatly increased and its side effects can be deeply reduced. Targeted drug delivery therapy is a new treatment method that has been widely used in various tumors, such as urothelial carcinoma, breast cancer, non-small cell lung cancer, and so on [16,17,18]. The study of kidney-targeted drug delivery began in 1990, and the concept was comprehensively proposed by Haas in 2002 [19]. During these two decades, various studies on kidney-targeted drug delivery have been published. It is well known that the kidney consists of basic renal units, which are composed of glomeruli and tubules, and the main pathological changes in DKD are glomerulosclerosis, tubular dysfunction, and tubulointerstitial fibrosis [20,21,22]. Therefore, the targeted treatment of DKD to glomeruli and tubules is of great significance. In current studies, the main focus is on glomerular podocytes, glomerular mesangial cells, and proximal tubular cells [23]. The purpose of this paper is to provide a comprehensive and important update on the development of drug delivery strategies for this target in proximal tubular epithelial cells, to summarize the rationale of its targeting and the associated drug carriers, in order to find new methods for the treatment of DKD.

## 2. Renal Proximal Tubular Cells

Targeted drug delivery to proximal tubular cells not only significantly enhances the efficiency of drug effects but also extremely reduces the negative side effects caused by drug action to other sites. This provides a new idea for the treatment of pathological changes such as tubulointerstitial fibrosis caused by DKD. In order to understand the targeted drug delivery to proximal tubular cells, we must first understand the physiological characteristics of the proximal tubule, its relationship with DKD, and its mechanism of drug uptake.

### 2.1. Physiological Characteristics of Proximal Tubule Cells

The kidney consists of basic renal units, which are composed of glomeruli and tubules. The renal tubule consists of a single layer of epithelial cells arranged on the proximal tubule, distal tubule, and collecting duct, which undertakes the exchange of substances between blood and urine. Therefore, the main physiological functions of renal tubules are reabsorption and secretion [24]. The proximal tubule plays a primary role in reabsorption, participating in the reabsorption of approximately two-thirds of the filtrate and recovering many compounds from the urine [25]. In addition, proximal tubules also have a potent secretory role, especially activated proximal tubular epithelial cells, which can promote the formation and development of tubulointerstitial lesions, reduced renal function, interstitial inflammation, and fibrosis through chemotaxis, antigen presentation, and cytokine autocrine and paracrine patterns [26,27]. This is very closely related to the development of DKD.

### 2.2. Relationship with DKD Progression

Currently, the most significant research on diabetes kidney disease has been focused on the glomerulus-centered research model. The pathological changes of the glomerulus, such as glomerular basement membrane thickening, mesangial expansion, increased resistance of endothelial cell fenestrations and podocyte injury [28,29,30,31], are the main pathological changes of DKD and are closely related to proteinuria, which is the signature of DKD. However, it has been shown that some patients with advanced disease show neither substantial glomerulopathy nor proteinuria but demonstrate a decline in traditional indicators of renal function (such as the presence of microalbuminuria or a decrease in glomerular filtration rate) [32]. Thus, structural and functional alterations of the renal tubules have an irreplaceable role in the progression of DKD. Renal tubular injury, mostly in the proximal tubule, is associated with abnormal activation of the AGEs-RAGE signaling pathway and is closely related to tubulointerstitial fibrosis, interstitial inflammation, and decreased renal function [33]. The mechanism that causes its injury is that due to the dependence of the proximal tubule itself on high energy and aerobic metabolism, when ischemic injury occurs in diabetic patients, increased consumption, impaired utilization, and reduced delivery of oxygen lead to apoptosis and fibrosis in the renal unit of the proximal tubule [34].

### 2.3. Mechanism of Proximal Tubule Uptake of Drugs

Although the renal tubules consist of proximal tubules, distal tubules, and collecting ducts, most tubule-targeting systems are aimed at the proximal tubules [35]. According to the anatomy of the kidney, drugs have to travel from the blood circulation to the proximal tubular cells, and there are two anatomical barriers between them, glomerular filtration barriers and basolateral barriers [36]. The glomerular filtration barrier is a highly specialized capillary wall that consists of three parts, including endothelial cells, podocytes, and basement membrane [37]. First, glomerular endothelial cells have the function of window opening, and the diameter of their window opening is approximately 70–100 nm [38]. There is an endothelial surface layer consisting of a membrane-bound layer of proteoglycans, glycosaminoglycans, and salivary proteins, a glycocalyx, and a loosely attached endothelial cell shell. Since salivary proteins, sulfated glycosaminoglycans, and hyaluronic acid in the endothelial surface layer are negatively charged, positively charged carriers pass through more easily [39]. Second, the glomerular basement membrane is composed of laminins, collagen IV, nidogens, and the heparan sulfate proteoglycans agrin, perlecan, and collagen XVIII. GBM is negatively charged due to the presence of negatively charged heparan sulfate proteoglycans agrin, which facilitates the passage of positively charged carriers [40]. Third, the podocyte is the terminally differentiated epithelial cell of the glomerulus, and its structure is traditionally divided into three distinct subcellular compartments: the cell body, the microtubule-driven membrane-extended primary process, and the actin-driven membrane-extended peduncle [41,42]. Adjacent podocyte cross each other at their foot processes, which are also called the slit diaphragm. The size of these slit diaphragms determines the size of the carrier that can pass through, with a passable carrier diameter of approximately 5–7 nm [43]. The basolateral barrier consists of the periportal capillary endothelium and the tubular mesenchyme between the capillaries and the proximal tubular cells. In order to cross this barrier, it first depends on the windowing of the peritubular capillary endothelium, which has a diameter of approximately 60–70 nm [44]. These opening windows are covered by a septum of approximately 3–5 nm thickness, which is composed of radial fibrils. The pore size to pass through these fibers is approximately 5.0–5.5 nm [45,46,47]. The surface layer of peritubular capillary endothelial cells contains negatively charged heparan sulfate so that positively charged drug carriers pass through more easily than negatively charged ones [48,49].

As the above description shows, it is easy to know that positively charged drugs with sizes smaller than 5–7 nm can reach proximal tubule cells from the bloodstream relatively easily and smoothly through the anatomical barrier. However, there are two ways that the drugs are taken up by the proximal tubule, one is the transporter protein, and the other is the receptor-mediated endocytosis [50]. The SLC subgroup is the uptake of drugs following chemical gradient-mediated passive transport. The ABC family is the uptake of drugs relying on ATP-depleted active transport [51,52]. Although there are so many transporter proteins, the feasibility of choosing them as transporters for targeted drug uptake is not high. First, as they are mainly involved in the uptake of small endogenous molecules and small molecules, targeted drugs with carriers may be too large for them. Second, the drug that is transported in by a transporter protein on one side of the proximal tubule may be quickly transported out again by the same or a different transporter protein on the other side. This may make it difficult to achieve effective drug concentrations in the proximal tubule. Finally, these transport proteins are widely distributed not only in the kidney but also in other organs [53,54]. This is the most limiting point for its application, because the most important thing for our targeted therapy is to increase the concentration of the drug in the kidney and reduce its side effects in other sites.

**Figure 1:**
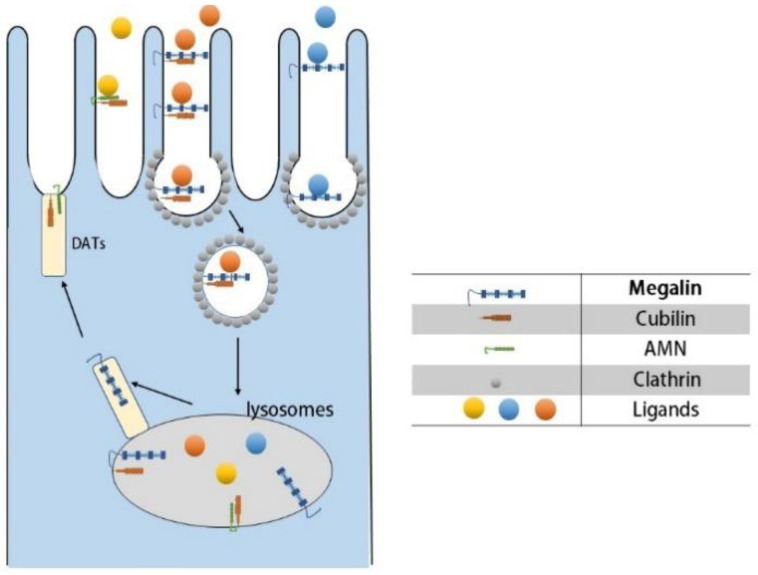
Receptor-mediated endocytosis of proximal tubules.

Consequently, we have to focus on receptor-mediated endocytosis (Figure 1). There are four receptors associated with targeted therapy, namely: megalin, cubilin, amnionless(AMN), and folate receptors [35]. Megalin and cubilin are present in the parietal membrane of proximal tubule epithelial cells, and it was shown that megalin cooperates with cubilin to facilitate the reabsorption of almost all filtered plasma proteins [55,56]. Megalin, as a multispecific clearance receptor, has a large extracellular structural domain, a single transmembrane structural domain, and a small cytoplasmic structural domain. In contrast, cubilin has only one extracellular structural domain and lacks the transmembrane and cytoplasmic structural domains, and it must interact with other membrane proteins for endocytosis. AMN has an extracellular structural domain, transmembrane structural domain, and cytoplasmic structural domain, and its main role is to assist the endocytosis of cubilin [57,58]. Based on differences in cellular ultrastructure, the proximal tubule is anatomically divided into three distinct segments S1 to S3, and it was shown that the s1 segment is highly specialized for receptor-mediated endocytosis [59,60]. Therefore, it is not difficult to conclude that the s1 fragment is the primary location where our targeted therapeutic drug is taken up by the proximal tubule. Megalin and cubilin combine and mediate endocytosis of a large and highly diverse set of ligands, including plasma proteins, peptides, enzymes, vitamin-binding proteins, hormones, and hormone-binding proteins, as well as drugs and toxins. Even though these two receptors have a wide range of ligands, their interaction with the ligands is specific [61]. As for the folate receptor (FR), its main role is to reabsorb folate from the renal tubular lumen. Among the four folate receptor subtypes identified in humans, the membrane-associated folate receptors FRα and FRβ were detected only in proximal renal tubule cells, which have a high specificity. In addition, folate receptors are overexpressed in a variety of malignant tissues and have been used for tumor-targeted delivery of anticancer drugs [62,63]. In summary, receptor-mediated endocytosis will be an important mechanism for our proximal tubule-targeted therapy.

(A) Ligand–receptor binding: Cubilin, due to its lack of transmembrane and cytoplasmic structural domains, must form a dual receptor complex with megelin or amnionless (AMN) for endocytosis to occur. Different ligands bind to the corresponding receptors with different affinities.
(B) Vesicle transport: After receptor–ligand binding, lattice proteins wrap the vesicles and transport them to the corresponding organelles (e.g., lysosomes) for further processing.
(C) Receptor recycling: After vesicle release of ligand, the receptor is recycled to the cell membrane by the apical tubules (DATs).

## 3. Targeted Drug Delivery Strategy

As previously discussed, the glomerular filtration barrier requires drugs smaller than 5–7 nm in diameter and positively charged to cross the barrier and reach the proximal tubule. Carriers that can be used as proximal tubule cell-targeted therapeutics include common proteins and peptides, polymers, small molecule prodrugs, and nanoparticles. I will summarize and discuss the applications and feasibility of these carriers in detail in the following sections.

### 3.1. Protein-Based and Peptide-Based Carriers

Low molecular weight proteins are the most widely researched carriers, which reach renal tubular cells via receptor-mediated endocytosis. The most popular of the low-molecular-weight proteins is lysozyme, with a molecular weight of approximately 14 kDa, which freely passes through the glomerular basement membrane and is endocytosed into proximal tubular cells expressing the giant protein receptor. Haas et al. [64] used lysozyme as a carrier to wrap naproxen targeted to the proximal tubule, resulting in a significant increase in naproxen accumulation in the kidney of approximately 70-fold. Dolman et al. [65] linked sunitinib analogs to lysozyme via a platinum-based linker to target them to the proximal tubule. The area of subcurvilinear renal drug levels was increased by 28-fold compared to equimolar doses of sunitinib malate, and its toxicity was substantially reduced. In spite of the fact that lysozyme is widely studied, the carrier itself can cause adverse effects such as systemic blood pressure and adverse effects on renal function [66]. In addition to lysozyme, another low molecular weight protein is the human serum protein fragment. Vegt et al. [67] first suggested in 2008 that the serum albumin fragment could be targeted to the kidney. Z.-x. Yuan et al. [68] used human serum albumin as the starting material, cleaved it into albumin fragments, and separated and purified the degradation products using Superdex 75 and CM-Sepharose FF to obtain three peptide fragments with specific sequences (PF-A_1–123_, PF-A_124–298_, and PF-A_299–585_). By studying their nephrotoxicity and cellular uptake, it was concluded that the human serum protein fragments not only showed good targeting (with PF-A_299–585_ being the best) but also no nephrotoxicity despite concentrations up to 5.00 mg/mL. Furthermore, in their next study [69], they applied PF-A299–585 as a vector to carry the Chinese herbal medicine rehmannia methylestradiol, which successfully targeted the kidney and alleviated the symptoms in a rodent model of membranous nephropathy. This certainly indicates that human serum protein fragment is an effective targeting carrier. Moreover, other proteins that can be reabsorbed by renal tubules, such as streptavidin [70] and somatostatin [71], also have some potential to become proximal tubule carriers. Thus, low molecular weight proteins have a bright and wide application prospect as targeting carriers for proximal tubule cells.

### 3.2. Polymeric Carriers

A number of studies have demonstrated that polymers can be used as carriers for proximal tubule targeting. The ability of polymers to accumulate in the renal tubules depends primarily on the type of anionic group, the copolymer monomer content, and the molecular weight of the final polymer. For example, low molecular weight polyvinylpyrrolidone (PVP) is excreted in the urine and does not accumulate in the kidney. However, anionized polyvinylpyrrolidones can remain in the kidney and carboxylated PVP exhibits higher renal accumulation than sulfonated PVPs. Kodeira et al. [72] found that carboxylated PVP accumulated in the kidney at 30% of the injected dose 3 h after administration, and most carboxylated PVP accumulated in the proximal tubule. On the basis of PVP, another PVP copolymer, poly (vinylpyrrolidone-co-dimethyl maleic anhydride) co-polymer (PVD), which has a higher renal accumulation, was investigated. Kamada et al. [73] found that approximately 80% of the 10-kDa PVD selectively accumulated in the kidney 4 h after intravenous administration of PVD to mice. Moreover, approximately 40% remained in the kidney 96 h after treatment. Yamamoto et al. [74], who used PVD as a carrier for the protein drug superoxide dismutase (SOD), showed that approximately six times more L-PVD-SOD than natural SOD was distributed to the kidney 3 h after intravenous injection.

Another targeting carrier that has been extensively studied is acetylated low molecular weight chitosan (LMWC). Using fluorescence imaging, Z.-X. Yuan et al. [75] found that random 50% N-acetylated low molecular weight chitosan (LMWC) selectively accumulated in the kidney, especially in the proximal tubule, and used LMWC as a carrier to piggyback on prednisolone and showed that the binding with a molecular weight of 19 kD had the highest accumulation rate in the kidney, and the total amount in the kidney was 13 times higher than that in the control prednisolone group D.-W. Wang et al. [76] constructed stepwise targeted chitosan triphenylphosphine-low molecular weight chitosan-curcumin (TPP-LMWC-CUR, TLC) and TLC in kidney tissue, causing rapid preferential distribution followed by specific internalization by renal tubular epithelial cells via interaction of the megalin with LMWC. LMWC is now well established for the targeted treatment of AKI and hyperuricemic kidney stones [76,77]; therefore, LMWC should have great potential as a vehicle for the treatment of DKD.

A recently emerging research trend is the use of various amino-acid-modified polyamide amine dendrimers as targeting carriers, which have been extensively investigated in tumors as well as osteoporosis [78,79]. Matsuura et al. [80] used L-serine (Ser)-modified polyamide amine dendrimers (PAMAM) as effective kidney-targeting drug carriers and showed that 3 h after intravenous injection, approximately in contrast, unmodified PAMAM, L-threonine-modified PAMAM, and L-tyrosine-modified PAMAM resulted in 28%, 35%, and 31% renal accumulation, respectively. The results suggest that Ser modification is a promising approach for renal targeting using macromolecular drug carriers.

Other carriers with proximal tubule targeting potential have been reported in the literature. For example, poly(N-(2-hydroxypropyl) methacrylamide) (pHPMA), which was applied for targeted tumor therapy, is currently being used [81,82]. In a study of the distribution of pHPMA in tumor-bearing mice, Kissel et al. [83] found a 33-fold higher concentration of biotin-pHPMA than HPMA in proximal tubular cells of both kidneys by day 7 after intravenous injection. This suggests that biotinylated pHPMA has the potential to target the kidney. However, its application is limited by the non-degradable nature of its backbone [84].

Similarly, poly-l-glutamic acid (PG) is widely used as a carrier for the delivery of anticancer chemotherapeutic drugs [85,86]. H.-J. Chai et al. [87] administered 3H-deoxycytidine-labeled PG and 3H-deoxycytidine intravenously to normal and streptozotocin-induced diabetic rats. The results showed that in normal rats, the renal accumulation was 7- or 15-fold higher in the group injected with PG carrier conjugates than in the non-injected group at 24 h after injection. In the kidneys of diabetic rats, the PG conjugate injected group was 8-fold higher than the control group. Additionally, after 24 h of injection, PG can be selectively deposited in the renal tubular epithelium. This study demonstrates the favorable accumulation properties of PG in normal and oxidative stress-induced kidneys with the potential for proximal tubule-targeted drug carriers. With the advancement of technology, more and more polymers are being invented, and polymer carriers have strong application prospects.

### 3.3. Small-Molecule Prodrugs

Prodrugs are simple chemical derivatives that require one or two enzymatic or chemical transformations to produce an active drug [88]. Large molecule drugs such as proteins often encounter significant barriers to penetration, so small molecule prodrugs are urgently needed. Folic acid, a carrier that has long been widely used in tumor-targeted therapy [89,90,91], works on the principle that tumor cells express folate receptors. Normal renal proximal tubules also express several folate receptors, so folic acid can be used to target drugs to proximal renal tubular cells [92]. When Trump et al. [93] labeled folic acid conjugates with radionuclides to observe their distribution at the tumor site, they unexpectedly found a high initial uptake of radionuclides in the proximal tubules of the kidney (17% ID/g at five minutes), which increased to 48% ID/g after 4 h. A noteworthy point is that folate receptors are expressed not only in proximal tubules but also in activated macrophages/monocytes and in organs other than the kidney (e.g., liver) [94]. Therefore, folic acid is controversial as a carrier for proximal-tubule-targeted therapy. Hyaluronic acid (HA) is also a small molecule prodrug, and Hu et al. [95] developed an HA-curcumin (HA-CUR) polymeric prodrug targeting proximal tubular epithelial cells. The biodistribution results showed a 13.9-fold increase in the accumulation of HA-CUR in the kidney compared to free CUR. This indicates that HA has a high prospect for proximal tubule targeting applications. There is also a small molecule prodrug called glycosylated small molecule prodrug. Lin et al. [96] designed carbamate-glucosamine conjugate (PCG), a combination of 2-aminoglucose and prednisolone, to ascertain the renal targeting ability of 2-aminoglucose. The results were poor tissue-specific localization of prednisolone and selective accumulation of PCG in the kidney with an 8.1-fold increase in drug concentration in the kidney 60 min after intravenous administration. The specific structural modification of the parent drug is key to the development of renal-targeted prodrugs [97].

### 3.4. Nanoparticles

Nanoparticles are a popular research topic nowadays. Nanoparticles are defined as particles less than 100 nm in diameter. Nanoparticles include: nanoliposomes, solid lipid nanoparticles, inorganic nanoparticles, organic nanoparticles, and micelles [98,99]. Because of their distinctive physical properties, such as surface effect and volume, in the past few years, nanoparticles have been successfully applied in many fields of medicine, such as cancer, diabetes, osteoarthritis, etc. [100,101,102]. The kidney has always been an important area for nanoparticle applications as well, not only because of the important role of the kidney in physiology but also because of the important role of the kidney in the filtration and excretion of nanoparticles [103]. Therefore, targeting nanoparticles to the renal tubules for the treatment of diabetic nephropathy is also very prospective. Whether the nanoparticles can reach the specified cellular destination depends on the size, homogeneity, surface potential, and drug loading of nanoparticles [104]. In the following, we detail the studies concerning these nanoparticles targeting the renal tubules.

First, nanoliposomes are an ideal and safe form of drug delivery with a characteristic lipid bilayer similar to the cytoplasmic membrane. They can incorporate a wide range of drug candidates in their hydrophilic and hydrophobic compartments [105]. Nanoliposomes can be administered by a variety of routes, including intravenous, transdermal, subcutaneous, or inhalation [106]. Nanoliposomes already have applications in Alzheimer’s disease, osteoporosis, and cancer, as well as promising applications in the treatment of kidney disease [107,108,109]. In a study by C. Huang et al. [110] they have prepared calycosin-loaded nanoliposomes for the treatment of diabetic nephropathy. The particle size, zeta potential, drug loading, and entrapment efficiency of this microparticle were 80 nm, −20.53 ± 3.57, 7.48 ± 1.19%, and 88.37 ± 2.28%, respectively. They used calycosin, an antioxidant, that plays a key role in reducing oxidative stress damage in kidney cells. As we mentioned before, oxidative stress is a key mechanism for tubular damage in diabetic nephropathy. They found that calycosin-loaded nanoliposomes not only reduced the first-pass effect of calycosin to improve its oral absorption but also prolonged its mean bioavailability to provide a slow release.

Second, the concept of solid lipid nanoparticles was first introduced in the early 1990s and has developed rapidly during the last three decades [111]. Solid lipid nanoparticles were investigated intensively as drug delivery systems for a number of delivery pathways, such as oral, parenteral, dermal, and topical delivery [112,113]. In contrast to nanoliposomes, solid lipid nanoparticles are devoid of cavities made up of bilayers. Ahangarpour et al. [114,115,116] utilized solid lipid nanoparticles of myricitrin, which has poor bioavailability, to successfully improve oxidative stress in proximal tubules induced by hyperglycemia. The microparticles they designed were spherical in shape with an average particle size, zeta potential, encapsulation efficiency, and encapsulation capacity of 76.1 nm, −5.51 mV, 56.2%, and 5.62% respectively. It is definitely exciting news for us to target proximal tubule cells for the treatment of DKD. In addition, in a work by M. H. Asfour et al. [117], they ingeniously designed all-trans retinoic acid loaded chitosan/tripolyphosphate lipid hybrid nanoparticles to treat DKD. This is a chitosan-coated solid lipid nanoparticle encapsulated with all-trans retinoic acid, which can greatly increase the oral availability of the drug. All-trans retinoic acid can protect renal tubules in the progression of DKD by inhibiting fibrosis [118]. They have produced spherical microparticles with a size of 338.26 nm and an encapsulation efficiency of 94.19%. Furthermore, when designing the solid nanoparticles, they have applied the characteristic surface effect of nanoparticles to increase the surface area and solubility of the drug. 

Third, as for organic nanoparticles, Liu et al. [119] wrapped Chinese medicine monomer Gypenoside (Gyp) XLIX with polymeric nanoparticles to form polylactic acid-co-glycoside (PLGA)-Gyp XLIX nanoparticles. Particle size, zeta potential, encapsulation rate, and drug loading capacity of the microparticles, which they prepared by applying nanoprecipitation and nano-self-assembly methods, were 128 ± 5.89 nm, −35.6 ± 3.18 mV, 82.4 ± 5.36%, and 9.04 ± 0.76%, respectively. The microparticles they prepared targeted the kidney and effectively inhibited tubular fibrosis and tubular necrosis, which are pathological changes found in DKD. Oroojalian et al. [120] used polymyxin-polyetherimide/DNA nanoparticles formulated with polymyxin B to successfully target proximal tubule cells expressing the megalin and demonstrated that it has higher transfection efficiency and lower cytotoxicity than unmodified PEIs/DNA nanoparticles. The microparticles sizes and zeta potentials prepared were 143–180 nm and 16.4 ± 1.87 to 23.43 ± 1.25 mV, correspondingly.

Fourth, inorganic nanoparticles, such as Fe_3_O_4_ magnetic nanoparticles, attenuated renal tubular injury and effectively inhibited tubular fibrosis with Fe_3_O_4_ magnetic albumin nanoparticles in a study by Liu et al. [121]. The hydrodynamic size and zeta potential of these microparticles were 102 nm and −18.6 mV, respectively. The intelligent use of albumin-coated iron particles effectively prolonged the in vivo circulation time of the particles and reduced off-target side effects [122].

Finally, Micelles are nanoparticles in the size range of 10–100 nm formed by the polymerization of monomers consisting of a hydrophilic head group and a hydrophobic tail group. J. Wang et al. [123] used micelles linked to kidney-targeting peptide (Lys-Lys-Glu-Glu-Glu)3-Lys) to successfully target the megalin on renal tubular epithelial cells. The average diameter of these particles was 15.0 ± 0.0 nm with the zeta potential of −7.8 ± 0.5 mV.

On the other hand, mesoscale nanoparticles refer to the larger gamut of nanoparticles above 100 nm in diameter. In the study by Williams et al. [124,125], they prepared a type of mesoscale nanoparticles with a diameter of approximately 400 nm that were formed from poly (lactic-co-glycolic acid) conjugated to polyethylene glycol, and observed that the nanoparticles could selectively and stably localize to the renal proximal tubular epithelial cells in experimental animals by the fluorescent tracer method. This microparticle size and surface charge were 347.6 ± 21.0 nm and −19.0 ± 0.3 mV, respectively. Their studies found that surface PEGylation facilitated particle localization to the kidney, long-term degradation, and controlled payload release. Moreover, they found that their localization efficiency in the kidney was 26–94 times higher than that in other organs, and they specifically targeted the renal tubules, and they had no inhibitory effect on renal function and no systemic toxicity. This carrier particle has already been successfully applied to encapsulate the selective TLR9 antagonist ODN2088 renal tubular targeting to treat tubular necrosis, inflammation, and apoptosis. This certainly encourages its application for therapeutic studies in DKD [126].

In addition, nanomaterials are not only nanoparticles but also nanotubes, nanocapsules, nanofibers and nanofilms. The tunneling nanotube is a channel that connects cells over long distances and has a diameter of 20–500 nm. The tunneling nanotube-TNFAIP2/M-sec system is used to treat autophagy of podocytes in DKD [127]. From this, we can look forward to future studies to discover whether new proteins present in the renal tubules are involved in the formation of tunneling nanotubes for the targeted treatment of DKD. Nanocapsules are also a common nanomaterial that has great potential in the therapy of DKD. In a study by M. Yu et al. [128], they used reactive-oxygen-species-responsive nanocapsules wrapped around adropin to effectively control blood glucose and lipid levels in DKD mice, inhibit the production of reactive oxygen species, and alleviate oxidative stress in DKD.

## 4. Conclusions and Future Perspectives

Drug targeting to proximal renal tubular cells is an increasingly sophisticated technique with great potential for development. The proximal tubular cell is a very promising targeting cell because it can transport and accumulate carriers into proximal tubular cells via receptor-mediated endocytosis to achieve appropriate drug concentrations and reduce drug side effects. We summarize four types of carriers that can target proximal tubule cells, including protein- and peptide-based carriers, polymeric carriers, small-molecule prodrugs, and nanoparticles. (Table 1) Among them, nanoparticles may be the most promising carriers for future clinical practice. Compared with the other three carriers, it has lower nephrotoxicity than protein and peptide carriers, better properties than polymer carriers with almost no renal excretion, and better targeting efficiency and retention than small molecule pre-drugs. Nanoparticles have their own unique nano-effects, such as surface effects that can greatly increase the surface area of the encapsulated drug to increase its absorbability. At present, the point that needs to be overcome for nanoparticle carriers is whether mass production and more precise targeting can be achieved. Nanotechnology is developing rapidly today, with technologies such as nanoenzymes, atomic force microscopes, and nanochips making a splash in various fields. We believe that with the future expansion of the field of nanotechnology, it is hopeful that nanoparticles with the ability to mass-produce and precisely target proximal renal tubular cells will be developed to slow down the progression of DKD and cur more and more patients with DKD.

## Figures and Tables

**Table 1:** Drug carriers targeting proximal tubule cells.

Carriers	Applications	Limitations
Protein-based and peptide-based carriers	Lysozyme	Systemic hypotension and nephrotoxicity
	Albumin fragment	
	Streptavidin	
	Somatostatin	
Polymeric carriers	PVP	Inferior biodegradability
	PVD	
	LMWC	
	PAMAM	
	pHPMA	
	PG	
Small-molecule prodrugs	Folic acid	Low targeting efficiency, retentiveness, and poor cell permeability
	Hyaluronic acid	
	PCG	
Nanoparticles	Nanoliposomes	Difficulty in mass production and lack of technology
	Solid lipid nanoparticles	
	Organic nanoparticles	
	Inorganic nanoparticles	
	Micelles	

## Data Availability

Not applicable.

## References

[1] Chen Z., Zhang W., Chen X., Hsu C.-Y. (2019). Trends in end-stage kidney disease in Shanghai, China. Kidney Int..

[2] Wang J., Zhang L., Tang S.C.-W., Kashihara N., Kim Y.-S., Togtokh A., Yang C.-W., Zhao M.-H. (2018). Disease burden and challenges of chronic kidney disease in North and East Asia. Kidney Int..

[3] Deng Y., Li N., Wu Y., Wang M., Yang S., Zheng Y., Deng X., Xiang D., Zhu Y., Xu P. (2021). Global, Regional, and National Burden of Diabetes-Related Chronic Kidney Disease From 1990 to 2019. Front. Endocrinol..

[4] Pollock C., Stefánsson B., Reyner D., Rossing P., Sjöström C.D., Wheeler D.C., Langkilde A.M., Heerspink H.J.L. (2019). Albuminuria-lowering effect of dapagliflozin alone and in combination with saxagliptin and effect of dapagliflozin and saxagliptin on glycaemic control in patients with type 2 diabetes and chronic kidney disease (DELIGHT): A randomised, double-blind, placebo-controlled trial. Lancet Diabetes Endocrinol..

[5] Barrera-Chimal J., Lima-Posada I., Bakris G.L., Jaisser F. (2022). Mineralocorticoid receptor antagonists in diabetic kidney disease—Mechanistic and therapeutic effects. Nat. Rev. Nephrol..

[6] Mann J.F.E., Ørsted D.D., Brown-Frandsen K., Marso S.P., Poulter N.R., Rasmussen S., Tornøe K., Zinman B., Buse J.B. (2017). Liraglutide and Renal Outcomes in Type 2 Diabetes. N. Engl. J. Med..

[7] Cherney D., Perkins B.A., Lytvyn Y., Heerspink H., Rodríguez-Ortiz M.E., Mischak H. (2017). The effect of sodium/glucose cotransporter 2 (SGLT2) inhibition on the urinary proteome. PLoS ONE.

[8] Lytvyn Y., Bjornstad P., Van Raalte D.H., Heerspink H.L., Cherney D.Z.I. (2020). The New Biology of Diabetic Kidney Disease-Mechanisms and Therapeutic Implications. Endocr. Rev..

[9] Ravindran S., Munusamy S. (2022). Renoprotective mechanisms of sodium-glucose co-transporter 2 (SGLT2) inhibitors against the progression of diabetic kidney disease. J. Cell. Physiol..

[10] Zhang M., He L., Liu J., Zhou L. (2021). Luteolin Attenuates Diabetic Nephropathy through Suppressing Inflammatory Response and Oxidative Stress by Inhibiting STAT3 Pathway. Exp. Clin. Endocrinol. Diabetes.

[11] Chen Y., Yang Y., Liu Z., He L. (2022). Adiponectin promotes repair of renal tubular epithelial cells by regulating mitochondrial biogenesis and function. Metabolism.

[12] Liu Z., Liu H., Xiao L., Liu G., Sun L., He L. (2019). STC-1 ameliorates renal injury in diabetic nephropathy by inhibiting the expression of BNIP3 through the AMPK/SIRT3 pathway. Lab. Invest..

[13] Chen Y.-Y., Peng X.-F., Liu G.-Y., Liu J.-S., Sun L., Liu H., Xiao L., He L.-Y. (2019). Protein arginine methyltranferase-1 induces ER stress and epithelial-mesenchymal transition in renal tubular epithelial cells and contributes to diabetic nephropathy. Biochim. Biophys. Acta Mol. Basis Dis..

[14] Cheng L., Qiu X., He L., Liu L. (2022). MicroRNA-122-5p ameliorates tubular injury in diabetic nephropathy via FIH-1/HIF-1α pathway. Ren. Fail..

[15] Bae Y.H., Park K. (2020). Advanced drug delivery 2020 and beyond: Perspectives on the future. Adv. Drug. Deliv. Rev..

[16] Arbour K.C., Riely G.J. (2019). Systemic Therapy for Locally Advanced and Metastatic Non-Small Cell Lung Cancer: A Review. JAMA.

[17] Li Y., Wu J., Qiu X., Dong S., He J., Liu J., Xu W., Huang S., Hu X., Xiang D.-X. (2023). Bacterial outer membrane vesicles-based therapeutic platform eradicates triple-negative breast tumor by combinational photodynamic/chemo-/immunotherapy. Bioact. Mater..

[18] Kleinmann N., Matin S.F., Pierorazio P.M., Gore J.L., Shabsigh A., Hu B., Chamie K., Godoy G., Hubosky S., Rivera M. (2020). Primary chemoablation of low-grade upper tract urothelial carcinoma using UGN-101, a mitomycin-containing reverse thermal gel (OLYMPUS): An open-label, single-arm, phase 3 trial. Lancet Oncol..

[19] Haas M., Moolenaar F., Meijer D.K.F., de Zeeuw D. (2002). Specific drug delivery to the kidney. Cardiovasc. Drugs Ther..

[20] Vallon V., Thomson S.C. (2012). Renal function in diabetic disease models: The tubular system in the pathophysiology of the diabetic kidney. Annu. Rev. Physiol..

[21] Qian Y., Feldman E., Pennathur S., Kretzler M., Brosius F.C. (2008). From fibrosis to sclerosis: Mechanisms of glomerulosclerosis in diabetic nephropathy. Diabetes.

[22] Ponchiardi C., Mauer M., Najafian B. (2013). Temporal profile of diabetic nephropathy pathologic changes. Curr. Diab. Rep..

[23] Liu C.-P., Hu Y., Lin J.-C., Fu H.-L., Lim L.Y., Yuan Z.-X. (2019). Targeting strategies for drug delivery to the kidney: From renal glomeruli to tubules. Med. Res. Rev..

[24] Geng H., Lan R., Liu Y., Chen W., Wu M., Saikumar P., Weinberg J.M., Venkatachalam M.A. (2021). Proximal tubule LPA1 and LPA2 receptors use divergent signaling pathways to additively increase profibrotic cytokine secretion. Am. J. Physiol. Renal. Physiol..

[25] Stormark T.A., Strømmen K., Iversen B.M., Matre K. (2007). Three-dimensional ultrasonography can detect the modulation of kidney volume in two-kidney, one-clip hypertensive rats. Ultrasound. Med. Biol..

[26] Zou Z., Chung B., Nguyen T., Mentone S., Thomson B., Biemesderfer D. (2004). Linking receptor-mediated endocytosis and cell signaling: Evidence for regulated intramembrane proteolysis of megalin in proximal tubule. J. Biol. Chem..

[27] Christensen E.I., Gburek J. (2004). Protein reabsorption in renal proximal tubule-function and dysfunction in kidney pathophysiology. Pediatr. Nephrol..

[28] Dai H., Liu Q., Liu B. (2017). Research Progress on Mechanism of Podocyte Depletion in Diabetic Nephropathy. J. Diabetes Res..

[29] Finch N.C., Fawaz S.S., Neal C.R., Butler M.J., Lee V.K., Salmon A.J., Lay A.C., Stevens M., Dayalan L., Band H. (2022). Reduced Glomerular Filtration in Diabetes Is Attributable to Loss of Density and Increased Resistance of Glomerular Endothelial Cell Fenestrations. J. Am. Soc. Nephrol..

[30] Kim D., Li H.Y., Lee J.H., Oh Y.S., Jun H.-S. (2019). Lysophosphatidic acid increases mesangial cell proliferation in models of diabetic nephropathy via Rac1/MAPK/KLF5 signaling. Exp. Mol. Med..

[31] Zhang J., Wang Y., Gurung P., Wang T., Li L., Zhang R., Li H., Guo R., Han Q., Zhang J. (2018). The relationship between the thickness of glomerular basement membrane and renal outcomes in patients with diabetic nephropathy. Acta Diabetol..

[32] Krolewski A.S. (2015). Progressive renal decline: The new paradigm of diabetic nephropathy in type 1 diabetes. Diabetes Care.

[33] Haraguchi R., Kohara Y., Matsubayashi K., Kitazawa R., Kitazawa S. (2020). New Insights into the Pathogenesis of Diabetic Nephropathy: Proximal Renal Tubules Are Primary Target of Oxidative Stress in Diabetic Kidney. Acta Histochem. Cytochem..

[34] Gilbert R.E. (2017). Proximal Tubulopathy: Prime Mover and Key Therapeutic Target in Diabetic Kidney Disease. Diabetes.

[35] Christensen E.I., Birn H., Storm T., Weyer K., Nielsen R. (2012). Endocytic receptors in the renal proximal tubule. Physiology (Bethesda).

[36] Kaissling B., Hegyi I., Loffing J., Le Hir M. (1996). Morphology of interstitial cells in the healthy kidney. Anat. Embryol..

[37] Li A.S., Ingham J.F., Lennon R. (2020). Genetic Disorders of the Glomerular Filtration Barrier. Clin. J. Am. Soc. Nephrol..

[38] Akilesh S., Huber T.B., Wu H., Wang G., Hartleben B., Kopp J.B., Miner J.H., Roopenian D.C., Unanue E.R., Shaw A.S. (2008). Podocytes use FcRn to clear IgG from the glomerular basement membrane. Proc. Natl. Acad. Sci. USA.

[39] Sverrisson K., Axelsson J., Rippe A., Asgeirsson D., Rippe B. (2014). Dynamic, size-selective effects of protamine sulfate and hyaluronidase on the rat glomerular filtration barrier In Vivo. Am. J. Physiol. Renal. Physiol..

[40] Naylor R.W., Morais MR P.T., Lennon R. (2021). Complexities of the glomerular basement membrane. Nat. Rev. Nephrol..

[41] Sever S. (2021). Role of actin cytoskeleton in podocytes. Pediatr. Nephrol..

[42] Pavenstädt H., Kriz W., Kretzler M. (2003). Cell biology of the glomerular podocyte. Physiol. Rev..

[43] Schipper M.L., Iyer G., Koh A.L., Cheng Z., Ebenstein Y., Aharoni A., Keren S., Bentolila L.A., Li J., Rao J. (2009). Particle size, surface coating, and PEGylation influence the biodistribution of quantum dots in living mice. Small.

[44] Satchell S.C., Braet F. (2009). Glomerular endothelial cell fenestrations: An integral component of the glomerular filtration barrier. Am. J. Physiol. Renal. Physiol..

[45] Rabelink T.J., Wijewickrama D.C., De Koning E.J. (2007). Peritubular endothelium: The Achilles heel of the kidney?. Kidney Int..

[46] Shaw I., Rider S., Mullins J., Hughes J., Péault B. (2018). Pericytes in the renal vasculature: Roles in health and disease. Nat. Rev. Nephrol..

[47] Stan R.V., Kubitza M., Palade G.E. (1999). PV-1 is a component of the fenestral and stomatal diaphragms in fenestrated endothelia. Proc. Natl. Acad. Sci. USA.

[48] Alcorn D., Maric C., Mccausland J. (1999). Development of the renal interstitium. Pediatr. Nephrol..

[49] Bearer E.L., Orci L. (1985). Endothelial fenestral diaphragms: A quick-freeze, deep-etch study. J. Cell Biol..

[50] Ivanyuk A., Livio F., Biollaz J., Buclin T. (2017). Renal Drug Transporters and Drug Interactions. Clin. Pharmacokinet..

[51] Nigam S.K. (2018). The SLC22 Transporter Family: A Paradigm for the Impact of Drug Transporters on Metabolic Pathways, Signaling, and Disease. Annu. Rev. Pharmacol. Toxicol..

[52] Yaneff A., Sahores A., Gómez N., Carozzo A., Shayo C., Davio C. (2019). MRP4/ABCC4 As a New Therapeutic Target: Meta-Analysis to Determine cAMP Binding Sites as a Tool for Drug Design. Curr. Med. Chem..

[53] Koepsell H., Lips K., Volk C. (2007). Polyspecific organic cation transporters: Structure, function, physiological roles, and biopharmaceutical implications. Pharm. Res..

[54] El-Sheikh A.A.K., Masereeuw R., Russel F.G.M. (2008). Mechanisms of renal anionic drug transport. Eur. J. Pharmacol..

[55] Christensen E.I., Verroust P.J., Nielsen R. (2009). Receptor-mediated endocytosis in renal proximal tubule. Pflug. Arch..

[56] Christensen E.I., Birn H. (2002). Megalin and cubilin: Multifunctional endocytic receptors. Nat. Rev. Mol. Cell Biol..

[57] Coudroy G., Gburek J., Kozyraki R., Madsen M., Trugnan G., Moestrup S.K., Verroust P.J., Maurice M. (2005). Contribution of cubilin and amnionless to processing and membrane targeting of cubilin-amnionless complex. J. Am. Soc. Nephrol..

[58] Fyfe J.C., Madsen M., Højrup P., Christensen E.I., Tanner S.M., de la Chapelle A., He Q., Moestrup S.K. (2004). The functional cobalamin (vitamin B12)-intrinsic factor receptor is a novel complex of cubilin and amnionless. Blood.

[59] Carney E.F. (2019). Endocytosis in the proximal tubule. Nat. Rev. Nephrol..

[60] Hall A.M., Polesel M., Berquez M. (2021). The proximal tubule, protein uptake, and the riddle of the segments. Kidney Int..

[61] Leheste J.R., Rolinski B., Vorum H., Hilpert J., Nykjaer A., Jacobsen C., Aucouturier P., Moskaug J.O., Otto A., Christensen E.I. (1999). Megalin knockout mice as an animal model of low molecular weight proteinuria. Am. J. Pathol..

[62] Qu Y., Hao C., Zhai R., Yao W. (2020). Folate and macrophage folate receptor-β in idiopathic pulmonary fibrosis disease: The potential therapeutic target?. Biomed. Pharmacother..

[63] Liu J., Chen H., Liu Y., Shen Y., Meng F., Kaniskan H.Ü., Jin J., Wei W. (2021). Cancer Selective Target Degradation by Folate-Caged PROTACs. J. Am. Chem. Soc..

[64] Haas M., Kluppel A.C., Wartna E.S., Moolenaar F., Meijer D.K., de Jong P.E., de Zeeuw D. (1997). Drug-targeting to the kidney: Renal delivery and degradation of a naproxen-lysozyme conjugate In Vivo. Kidney Int..

[65] Dolman M.E.M., Harmsen S., Pieters E.H.E., Sparidans R.W., Lacombe M., Szokol B., Orfi L., Kéri G., Storm G., Hennink W.E. (2012). Targeting of a platinum-bound sunitinib analog to renal proximal tubular cells. Int. J. Nanomed..

[66] Haverdings R.F., Haas M., Greupink A.R., de Vries P.A., Moolenaar F., de Zeeuw D., Meijer D.K. (2001). Potentials and limitations of the low-molecular-weight protein lysozyme as a carrier for renal drug targeting. Ren. Fail..

[67] Vegt E., Van Eerd J.E.M., Eek A., Oyen W.J.G., Wetzels J.F.M., de Jong M., Russel F.G.M., Masereeuw R., Gotthardt M., Boerman O.C. (2008). Reducing renal uptake of radiolabeled peptides using albumin fragments. J. Nucl. Med..

[68] Yuan Z.-X., He X.-K., Wu X.-J., Gao Y., Fan M., Song L.-Q., Xu C.-Q. (2014). Peptide fragments of human serum albumin as novel renal targeting carriers. Int. J. Pharm..

[69] Yuan Z.-X., Wu X.-J., Mo J., Wang Y.-l., Xu C.-q., Lim L.Y. (2015). Renal targeted delivery of triptolide by conjugation to the fragment peptide of human serum albumin. Eur. J. Pharm. Biopharm..

[70] Schechter B., Arnon R., Colas C., Burakova T., Wilchek M. (1995). Renal accumulation of streptavidin: Potential use for targeted therapy to the kidney. Kidney Int..

[71] Reubi J.C., Horisberger U., Studer U.E., Waser B., Laissue J.A. (1993). Human kidney as target for somatostatin: High affinity receptors in tubules and vasa recta. J. Clin. Endocrinol. Metab..

[72] Kodaira H., Tsutsumi Y., Yoshioka Y., Kamada H., Kaneda Y., Yamamoto Y., Tsunoda S.-i., Okamoto T., Mukai Y., Shibata H. (2004). The targeting of anionized polyvinylpyrrolidone to the renal system. Biomaterials.

[73] Kamada H., Tsutsumi Y., Sato-Kamada K., Yamamoto Y., Yoshioka Y., Okamoto T., Nakagawa S., Nagata S., Mayumi T. (2003). Synthesis of a poly(vinylpyrrolidone-co-dimethyl maleic anhydride) co-polymer and its application for renal drug targeting. Nat. Biotechnol..

[74] Yamamoto Y., Tsutsumi Y., Yoshioka Y., Kamada H., Sato-Kamada K., Okamoto T., Mukai Y., Shibata H., Nakagawa S., Mayumi T. (2004). Poly(vinylpyrrolidone-co-dimethyl maleic acid) as a novel renal targeting carrier. J. Control. Release.

[75] Yuan Z.-X., Sun X., Gong T., Ding H., Fu Y., Zhang Z.-R. (2007). Randomly 50% N-acetylated low molecular weight chitosan as a novel renal targeting carrier. J. Drug. Target.

[76] Wang D.-W., Li S.-J., Tan X.-Y., Wang J.-H., Hu Y., Tan Z., Liang J., Hu J.-B., Li Y.-G., Zhao Y.-F. (2021). Engineering of stepwise-targeting chitosan oligosaccharide conjugate for the treatment of acute kidney injury. Carbohydr. Polym..

[77] Kandav G., Bhatt D.C., Singh S.K. (2022). Effect of Different Molecular Weights of Chitosan on Formulation and Evaluation of Allopurinol-Loaded Nanoparticles for Kidney Targeting and in Management of Hyperuricemic Nephrolithiasis. AAPS PharmSciTech.

[78] Ren M., Li Y., Zhang H., Li L., He P., Ji P., Yang S. (2021). An oligopeptide/aptamer-conjugated dendrimer-based nanocarrier for dual-targeting delivery to bone. J. Mater. Chem. B.

[79] Hu Q., Wang Y., Xu L., Chen D., Cheng L. (2020). Transferrin Conjugated pH- and Redox-Responsive Poly(Amidoamine) Dendrimer Conjugate as an Efficient Drug Delivery Carrier for Cancer Therapy. Int. J. Nanomed..

[80] Matsuura S., Katsumi H., Suzuki H., Hirai N., Hayashi H., Koshino K., Higuchi T., Yagi Y., Kimura H., Sakane T. (2018). l-Serine-modified polyamidoamine dendrimer as a highly potent renal targeting drug carrier. Proc. Natl. Acad. Sci. USA.

[81] Wang Y., Xia H., Chen B., Wang Y. (2022). Rethinking nanoparticulate polymer-drug conjugates for cancer theranostics. Wiley Interdiscip. Rev. Nanomed. Nanobiotechnol..

[82] Duan Z., Luo Q., Dai X., Li X., Gu L., Zhu H., Tian X., Zhang H., Gong Q., Gu Z. (2021). Synergistic Therapy of a Naturally Inspired Glycopolymer-Based Biomimetic Nanomedicine Harnessing Tumor Genomic Instability. Adv. Mater..

[83] Kissel M., Peschke P., Subr V., Ulbrich K., Strunz A.M., Kühnlein R., Debus J., Friedrich E. (2002). Detection and cellular localisation of the synthetic soluble macromolecular drug carrier pHPMA. Eur. J. Nucl. Med. Mol. Imaging.

[84] Kukowska-Latallo J.F., Candido K.A., Cao Z., Nigavekar S.S., Majoros I.J., Thomas T.P., Balogh L.P., Khan M.K., Baker J.R. (2005). Nanoparticle targeting of anticancer drug improves therapeutic response in animal model of human epithelial cancer. Cancer Res..

[85] Wang Y., Shen N., Wang Y., Li M., Zhang W., Fan L., Liu L., Tang Z., Chen X. (2021). Cisplatin nanoparticles boost abscopal effect of radiation plus anti-PD1 therapy. Biomater. Sci..

[86] Cheah H.Y., Gallon E., Dumoulin F., Hoe S.Z., Japundžić-Žigon N., Glumac S., Lee H.B., Anand P., Chung L.Y., Vicent M.J. (2018). Near-Infrared Activatable Phthalocyanine-Poly-L-Glutamic Acid Conjugate: Enhanced In Vivo Safety and Antitumor Efficacy toward an Effective Photodynamic Cancer Therapy. Mol. Pharm..

[87] Chai H.-J., Kiew L.-V., Chin Y., Norazit A., Mohd Noor S., Lo Y.-L., Looi C.-Y., Lau Y.-S., Lim T.-M., Wong W.-F. (2017). Renal targeting potential of a polymeric drug carrier, poly-l-glutamic acid, in normal and diabetic rats. Int. J. Nanomed..

[88] Mishra A.P., Chandra S., Tiwari R., Srivastava A., Tiwari G. (2018). Therapeutic Potential of Prodrugs Towards Targeted Drug Delivery. Open Med. Chem. J..

[89] Chen Z., Wang W., Li Y., Wei C., Zhong P., He D., Liu H., Wang P., Huang Z., Zhu W. (2021). Folic Acid-Modified Erythrocyte Membrane Loading Dual Drug for Targeted and Chemo-Photothermal Synergistic Cancer Therapy. Mol. Pharm..

[90] Qiu L., Dong C., Kan X. (2018). Lymphoma-targeted treatment using a folic acid-decorated vincristine-loaded drug delivery system. Drug Des. Dev. Ther..

[91] Lu Y., Low P.S. (2002). Folate-mediated delivery of macromolecular anticancer therapeutic agents. Adv. Drug Deliv. Rev..

[92] Low P.S., Kularatne S.A. (2009). Folate-targeted therapeutic and imaging agents for cancer. Curr. Opin. Chem. Biol..

[93] Trump D.P., Mathias C.J., Yang Z., Low P.S., Marmion M., Green M.A. (2002). Synthesis and evaluation of 99mTc(CO)(3)-DTPA-folate as a folate-receptor-targeted radiopharmaceutical. Nucl. Med. Biol..

[94] Yang Y., Guo L., Wang Z., Liu P., Liu X., Ding J., Zhou W. (2021). Targeted silver nanoparticles for rheumatoid arthritis therapy via macrophage apoptosis and Re-polarization. Biomaterials.

[95] Hu J.-B., Li S.-J., Kang X.-Q., Qi J., Wu J.-H., Wang X.-J., Xu X.-L., Ying X.-Y., Jiang S.-P., You J. (2018). CD44-targeted hyaluronic acid-curcumin prodrug protects renal tubular epithelial cell survival from oxidative stress damage. Carbohydr. Polym..

[96] Lin Y., Li Y., Wang X., Gong T., Zhang L., Sun X. (2013). Targeted drug delivery to renal proximal tubule epithelial cells mediated by 2-glucosamine. J. Control. Release.

[97] Yuan Z.-X., Shang Z., Gu J., He L. (2019). Renal targeting delivery systems. Future Med. Chem..

[98] Mukherjee A., Waters A.K., Kalyan P., Achrol A.S., Kesari S., Yenugonda V.M. (2019). Lipid-polymer hybrid nanoparticles as a next-generation drug delivery platform: State of the art, emerging technologies, and perspectives. Int. J. Nanomed..

[99] Mu H., Holm R. (2018). Solid lipid nanocarriers in drug delivery: Characterization and design. Expert Opin. Drug Deliv..

[100] Xu S., Chang L., Zhao X., Hu Y., Lin Y., Chen Z., Ren X., Mei X. (2022). Preparation of epigallocatechin gallate decorated Au-Ag nano-heterostructures as NIR-sensitive nano-enzymes for the treatment of osteoarthritis through mitochondrial repair and cartilage protection. Acta Biomater..

[101] Mei X., Hu T., Wang H., Liang R., Bu W., Wei M. (2020). Highly dispersed nano-enzyme triggered intracellular catalytic reaction toward cancer specific therapy. Biomaterials.

[102] Luo X.-M., Yan C., Feng Y.-M. (2021). Nanomedicine for the treatment of diabetes-associated cardiovascular diseases and fibrosis. Adv. Drug Deliv. Rev..

[103] Hauser P.V., Chang H.M., Yanagawa N., Hamon M. (2021). Nanotechnology, Nanomedicine, and the Kidney. Appl. Sci..

[104] Albanese A., Tang P.S., Chan W.C.W. (2012). The effect of nanoparticle size, shape, and surface chemistry on biological systems. Annu. Rev. Biomed. Eng..

[105] Filipczak N., Pan J., Yalamarty S.S.K., Torchilin V.P. (2020). Recent advancements in liposome technology. Adv. Drug Deliv. Rev..

[106] Paluszkiewicz P., Martuszewski A., Zaręba N., Wala K., Banasik M., Kepinska M. (2021). The Application of Nanoparticles in Diagnosis and Treatment of Kidney Diseases. Int. J. Mol. Sci..

[107] Chen C.-H., Weng T.-H., Chuang C.-H., Huang K.-Y., Huang S.-C., Chen P.-R., Huang H.-H., Huang L.-Y., Shen P.-C., Chuang P.-Y. (2022). Transdermal nanolipoplex simultaneously inhibits subcutaneous melanoma growth and suppresses systemically metastatic melanoma by activating host immunity. Nanomedicine.

[108] Salave S., Rana D., Kumar H., Kommineni N., Benival D. (2022). Anabolic Peptide-Enriched Stealth Nanoliposomes for Effective Anti-Osteoporotic Therapy. Pharmaceutics.

[109] Passeri E., Elkhoury K., Morsink M., Broersen K., Linder M., Tamayol A., Malaplate C., Yen F.T., Arab-Tehrany E. (2022). Alzheimer’s Disease: Treatment Strategies and Their Limitations. Int. J. Mol. Sci..

[110] Huang C., Xue L.-F., Hu B., Liu H.-H., Huang S.-B., Khan S., Meng Y. (2021). Calycosin-loaded nanoliposomes as potential nanoplatforms for treatment of diabetic nephropathy through regulation of mitochondrial respiratory function. J. Nanobiotechnol..

[111] Müller R.H., Shegokar R., Keck C.M. (2011). 20 years of lipid nanoparticles (SLN and NLC): Present state of development and industrial applications. Curr. Drug Discov. Technol..

[112] Subroto E., Andoyo R., Indiarto R., Wulandari E., Wadhiah E.F.N. (2022). Preparation of Solid Lipid Nanoparticle-Ferrous Sulfate by Double Emulsion Method Based on Fat Rich in Monolaurin and Stearic Acid. Nanomaterials.

[113] Iqbal M.A., Md S., Sahni J.K., Baboota S., Dang S., Ali J. (2012). Nanostructured lipid carriers system: Recent advances in drug delivery. J. Drug Target..

[114] Ahangarpour A., Oroojan A.A., Khorsandi L., Kouchak M., Badavi M. (2018). Solid Lipid Nanoparticles of Myricitrin Have Antioxidant and Antidiabetic Effects on Streptozotocin-Nicotinamide-Induced Diabetic Model and Myotube Cell of Male Mouse. Oxid. Med. Cell. Longev..

[115] Ahangarpour A., Oroojan A.A., Khorsandi L., Kouchak M., Badavi M. (2019). Antioxidant, anti-apoptotic, and protective effects of myricitrin and its solid lipid nanoparticle on streptozotocin-nicotinamide-induced diabetic nephropathy in type 2 diabetic male mice. Iran. J. Basic Med. Sci..

[116] Ahangarpour A., Oroojan A.A., Khorsandi L., Kouchak M., Badavi M. (2021). Hyperglycemia-induced oxidative stress in isolated proximal tubules of mouse: The in vitro effects of myricitrin and its solid lipid nanoparticle. Arch. Physiol. Biochem..

[117] Asfour M.H., Salama A.A.A., Mohsen A.M. (2021). Fabrication of All-Trans Retinoic Acid loaded Chitosan/Tripolyphosphate Lipid Hybrid Nanoparticles as a Novel Oral Delivery Approach for Management of Diabetic Nephropathy in Rats. J. Pharm. Sci..

[118] Sierra-Mondragon E., Rodríguez-Muñoz R., Namorado-Tonix C., Molina-Jijon E., Romero-Trejo D., Pedraza-Chaverri J., Reyes J.L. (2019). All-Trans Retinoic Acid Attenuates Fibrotic Processes by Downregulating TGF-β1/Smad3 in Early Diabetic Nephropathy. Biomolecules.

[119] Liu Q., Chen X., Kan M., Yang J., Gong Q., Jin R., Dai Y., Jin J., Zang H. (2021). Gypenoside XLIX loaded nanoparticles targeting therapy for renal fibrosis and its mechanism. Eur. J. Pharmacol..

[120] Oroojalian F., Rezayan A.H., Mehrnejad F., Nia A.H., Shier W.T., Abnous K., Ramezani M. (2017). Efficient megalin targeted delivery to renal proximal tubular cells mediated by modified-polymyxin B-polyethylenimine based nano-gene-carriers. Mater. Sci. Eng. C Mater. Biol. Appl..

[121] Liu L., Xu Q., Zhang L., Sun H., Ding F., Li Y., Chen P. (2021). FeO magnetic nanoparticles ameliorate albumin-induced tubulointerstitial fibrosis by autophagy related to Rab7. Colloids Surf. B Biointerfaces.

[122] Guo L., Luo S., Du Z., Zhou M., Li P., Fu Y., Sun X., Huang Y., Zhang Z. (2017). Targeted delivery of celastrol to mesangial cells is effective against mesangioproliferative glomerulonephritis. Nat. Commun..

[123] Wang J., Poon C., Chin D., Milkowski S., Lu V., Hallows K.R., Chung E.J. (2018). Design and in vivo characterization of kidney-targeting multimodal micelles for renal drug delivery. Nano Res..

[124] Williams R.M., Shah J., Ng B.D., Minton D.R., Gudas L.J., Park C.Y., Heller D.A. (2015). Mesoscale nanoparticles selectively target the renal proximal tubule epithelium. Nano Lett..

[125] Williams R.M., Shah J., Tian H.S., Chen X., Geissmann F., Jaimes E.A., Heller D.A. (2018). Selective Nanoparticle Targeting of the Renal Tubules. Hypertension.

[126] Han S.J., Williams R.M., D'agati V., Jaimes E.A., Heller D.A., Lee H.T. (2020). Selective nanoparticle-mediated targeting of renal tubular Toll-like receptor 9 attenuates ischemic acute kidney injury. Kidney Int..

[127] Barutta F., Bellini S., Kimura S., Hase K., Corbetta B., Corbelli A., Fiordaliso F., Bruno S., Biancone L., Barreca A. (2022). Protective effect of the tunneling nanotube-TNFAIP2/M-sec system on podocyte autophagy in diabetic nephropathy. Autophagy.

[128] Yu M., Wang D., Zhong D., Xie W., Luo J. (2022). Adropin Carried by Reactive Oxygen Species-Responsive Nanocapsules Ameliorates Renal Lipid Toxicity in Diabetic Mice. ACS Appl. Mater. Interfaces.

